# Changes in temporal and spatial gait coordination during the six‑minute walk test in people with multiple sclerosis

**DOI:** 10.1007/s11517-026-03678-x

**Published:** 2026-09-20

**Authors:** Giulia Casu, Elisabetta Lostia di Santa Sofia, Bruno Leban, Cristina Inglese, Giancarlo Coghe, Jessica Frau, Eleonora Cocco, Massimiliano Pau

**Affiliations:** 1 Department of Mechanical, Chemical and Materials Engineering, University of Cagliari, Cagliari, Italy; 2 Multiple Sclerosis Centre, ASL Cagliari, Cagliari, Italy; 3 Department of Medical Sciences and Public Health, University of Cagliari, Cagliari, Italy

**Keywords:** Gait, Kinematics, Spatio-temporal, Multiple sclerosis (MS), Cyclograms, Angle-angle diagrams, Symmetry

## Abstract

Although gait coordination impairments represent a critical aspect of walking dysfunction in multiple sclerosis (MS), their variation during prolonged walking remains relatively unexplored. This study investigated changes in gait coordination during the six-minute walk test (6MWT) by jointly analyzing temporal and spatial measures of inter-limb and inter-joint coordination. Sixty-five people with MS and 43 matched unaffected individuals performed the 6MWT while kinematic data were acquired using an inertial measurement unit–based motion capture system. Coordination was quantified using the Phase Coordination Index and cyclogram-based geometric features, computed on a minute-by-minute basis. Results showed that people with MS exhibited significantly impaired coordination compared with unaffected individuals across most metrics from the beginning of the test. While both groups demonstrated progressive changes over time, people with MS showed limited additional deterioration during prolonged walking, suggesting a ceiling effect related to baseline neuromotor impairment and reduced adaptive capacity. Inter-joint coordination changes were most evident at the knee–ankle joint. Temporal and spatial measures provided complementary insights, revealing joint- and domain-specific behaviors missed by single-metric approaches. These findings support multimodal coordination analysis to improve gait assessment and guide rehabilitation in MS.

**Graphical Abstract:**

**Figure:**
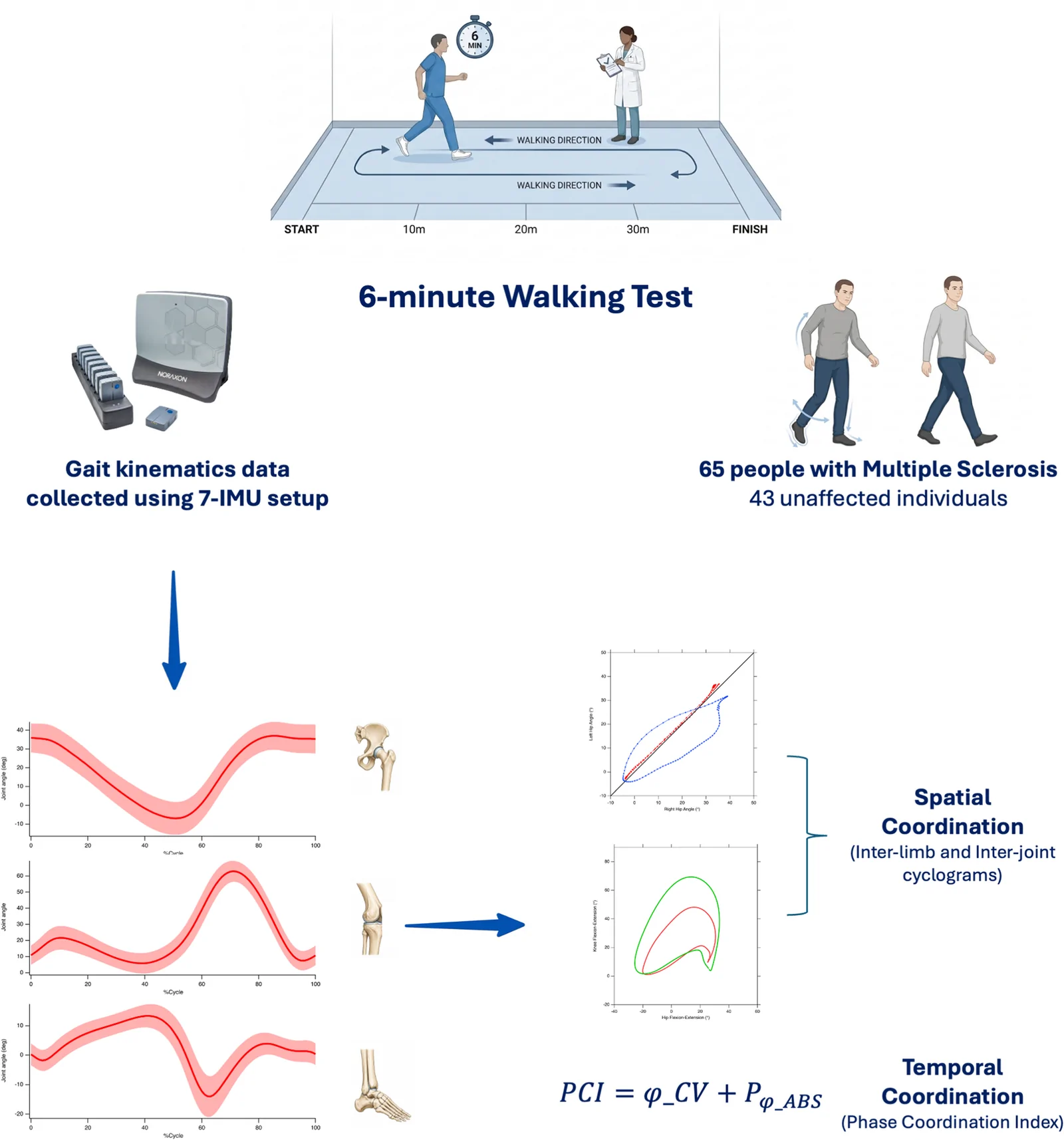

## Introduction

One of the most common and disabling consequences of Multiple Sclerosis (MS) is represented by impairment in ambulation, which manifests in terms of altered spatio-temporal, kinematic and kinetic parameters and affects approximately 70–75% of people with MS (pwMS) during the course of the disease [1]. Indeed, most studies that have been carried out in the last two decades using instrumental approaches (i.e., motion capture systems, electronic walkways, wearable inertial sensors, force platforms, etc.) allowed to depict a gait pattern characterized by peculiar features such as reduced speed, stride/step length and swing phase duration as well as increased duration of the double support phase [1, 2]. Similarly, when the sagittal kinematics is considered, pwMS show reduced hip extension during the stance phase, decreased peak of knee flexion during the swing phase as well as reduced dorsiflexion and decreased plantarflexion at the ankle joint respectively during the stance and pre-swing phase [1]. Such features, whose magnitude is mediated by disability level [3] and sex [4] are further exacerbated in presence of spasticity [5].

In contrast, relatively less attention has been devoted to aspects related to coordination, despite its recognized role in influencing gait quality and efficiency [6]. For the sake of accuracy, it should be acknowledged that this is not an issue specific to MS, but rather a common feature of most neurological conditions affecting locomotion. This likely reflects the challenges associated with establishing both a well-defined theoretical framework for this construct and standardized methods for its quantification. As highlighted by previous studies [7], this complexity arises from the context-dependent nature of locomotor coordination, which encompasses both spatial (i.e., relationships among the positions of different body segments or joints) and temporal (i.e., the relative timing of segment or joint positions throughout the motor task) components. A direct consequence of this multifaceted nature is the possibility of deriving both spatial and temporal metrics: the former are typically based on body segment or joint kinematics, whereas the latter focus on phase relationships between different body parts [7].

Over the past decade, several studies have examined pwMS using both approaches. The geometrical and mathematical features of angle–angle diagrams (known as “cyclograms” [8]) have been analyzed to quantify inter-limb and inter-joint coordination in pwMS and their ability to characterize gait impairments, fall history, fear of falling, and disability has subsequently been confirmed by several independent research groups [9–14].

These measures demonstrate strong discriminative capability, not only in distinguishing pwMS from unaffected individuals, but also in identifying differences across varying levels of disability within the pwMS population. Methods grounded in the concept of phase relationships between limb segments (i.e. Continuous Relative Phase, CRP) or events in the gait cycle (Phase Coordination Index, PCI) have also been successfully employed to quantify alterations of coordination in MS. Specifically, it has been shown that: (i) both CRP and PCI can detect coordination impairments in pwMS compared with unaffected individuals [9, 14–16]; (ii) a moderate significant association exists between coordination and disability level [17, 18]; and (iii) fatigue induced during a 6-minute walk test (6MWT) leads to a progressive deterioration in coordination [17]. Additionally, the analysis of lower-limb intersegmental coordination may offer further insight into fall risk in multiple sclerosis [14], whereas the potential use of coordination parameters to evaluate the effectiveness of aerobic exercise–based rehabilitation remains unclear [19].

Of particular interest is the possibility of investigating coordination changes associated with functional tests such as the 6MWT which, to date, has been explored in only a limited number of studies using exclusively PCI [17, 18, 20]. The 6MWT is a widely used and valuable tool for assessing functional exercise capacity in pwMS, as it provides an integrated measure of walking endurance reflecting the combined influence of motor function, cardiorespiratory capacity, and fatigue [21]. Moreover, it is able to effectively capture alterations in gait patterns associated with fatigue according to disability level [22] and suitable to detect changes over time or in response to rehabilitation interventions [23]. In addition, the 6MWT enables clinicians to evaluate walking fatigability by analyzing performance trends throughout the test [24], thereby offering insight into real-world mobility limitations and their impact on activities of daily living. What emerges from the existing literature is that coordination, expressed through PCI, deteriorates more during the 6MWT in those with higher disability, as expressed by the Expanded Disability Status Scale (EDSS) score [17], improves with increasing speed (i.e. fast vs. self-selected [18] and is characterized by moderate reliability depending on the considered moment of the test [20].

Such information, although valuable, describes only partly the possible changes in coordination associated with an endurance test like the 6MWT. Indeed, as previously mentioned, PCI is a temporal metric capturing the accuracy and variability of left–right stepping, which certainly provides important information on inter-limb timing, but does not capture how joint motions are spatially organized during gait. In this context, the use of cyclograms might offer important added value. In fact, as spatial measures of coordination, they describe the relationship between joint angles or between limbs across the gait cycle, thereby providing insight into the structure and quality of movement patterns. Specifically, cyclograms allow the assessment of inter-joint coordination (by quantifying coupling between adjacent segments and revealing alterations in motor control strategies), inter-limb coordination (through bilateral angle relationships) complementing temporal indices such as PCI as well as movement variability and consistency, via changes in shape, area, and repeatability across strides. Thus, the combined use of PCI and cyclograms during a 6MWT is therefore advantageous, as it enables a more comprehensive characterization of coordination impairments, particularly as in the case of MS, where neuromotor deficits may alter both the timing and the spatial organization of gait. Consequently, integrating spatial and temporal metrics may enhance sensitivity to subtle coordination deficits and improve the evaluation of disease progression and rehabilitation outcomes.

Based on these considerations, the present study intends to investigate existence and magnitude of possible changes in spatial and temporal measures of coordination during the 6MWT to verify: (1) if changes in coordination parameters, calculated on a minute-by-minute basis differs across pwMS and unaffected individuals, (2) whether spatial and temporal metrics share similar sensitivity in detecting alterations in coordination.

## Methods

### Participants

An a priori power analysis was conducted using G*Power version 3.1 (Heinrich Heine University, Düsseldorf, Germany) based on the primary outcome of the study, namely the PCI, as this metric was the most commonly employed to quantify inter-limb coordination during gait in pwMS. Estimates of the expected difference and variability were derived from previously published data [16, 17], who reported significantly higher PCI in pwMS compared with unaffected individuals and increasing PCI with higher levels of disability, indicating impaired gait coordination. Based on their data, a moderate effect size (Cohen’s d ≈ 0.5) was assumed. With an alpha level of 0.05 and a statistical power of 80%, the required sample size was estimated at 32 participants per group. To account for a potential dropout rate of approximately 15% (that is participants unable to complete the 6MWT), the target sample size was increased to 38 participants per group.

Based on such considerations, in the period December 2025 – April 2026, a total of 65 pwMS (49 women, 16 men, mean age 50.9 years) followed at the Regional Multiple Sclerosis Center of Sardinia (“R. Binaghi” Hospital, ASL Cagliari, Cagliari, Italy) were recruited for the study on a voluntary basis. They had previously been diagnosed with MS by a neurologist expert in MS (EC, GC, JF) according to the 2010 revised criteria [25, 26] and were able to ambulate autonomously (i.e., without the support of canes, crutches or walking frames) for at least 200 m (a distance consistent with existing data on 6MWT performance in pwMS with severe disability [27]) They were also free from other neurological or orthopedic condition potentially able to severely affect gait or balance. Forty-three unaffected individuals age- and sex-matched were recruited on a voluntary basis among nurses and staff of the MS Center and the University of Cagliari to serve as control group. None of the investigators had a supervisory or managerial role over them. The study was conducted according to the principles expressed in the World Medical Association Declaration of Helsinki and its later amendments and was approved by the Ethics Committee of the University of Cagliari (Prot. 0338891 of 12/18/2025). All participants signed an informed consent agreeing to participate. The main anthropometric and clinical features of all participants are reported in Table 1.

**Table 1:** Anthropometric and clinical features of participants. Values are expressed as mean (SD)

	Multiple Sclerosis	Unaffected individuals	t (Welch)	Two-sided *p*-value
Participants	65 (16 M, 49 F)	43 (11 M, 32 F)	-	-
F: M Ratio	3.06	2.91	-	-
Age (years)	50.9 (10.6)	46.9 (14.0)	−1.595	0.115
Body Mass (kg)	62.2 (12.1)	61.2 (9.9)	−0.496	0.620
Height (cm)	163.2 (9.0)	164.8 (7.4)	1.020	0.310
EDSS	2.9 (1.9)	-	-	-
Type of MS	55 RR, 3 PP, 7 SP	-	-	-

*EDSS*: Expanded Disability Status Scale; *MS*: Multiple Sclerosis; *PP*: Primary-Progressive; *RR*: Relapsing-Remitting; *SP*: Secondary-Progressive. Statistical analysis for anthropometric variables was carried out using Welch’s independent-samples t-test

### Experimental protocol for kinematic data collection

An IMU-based motion capture system (Ultium Motion, Noraxon Inc., Scottsdale, AZ, USA), composed of 8 miniaturized wearable sensors sampling at 200 Hz, was employed to acquire lower-limb and trunk accelerations and angular velocities. Sensors were positioned on the participant’s lower limbs and trunk, using dedicated straps, as follows: the lower spine sensor was placed In line with the spinal column approximately at L1/T12 vertebrae location, while the pelvic sensor on the sacrum; the thigh sensors on the anterior distal half of the femur; the tibial sensors on the medial surface of the proximal tibia and the forefoot sensors bilaterally on the dorsal aspect of the midfoot. Before each data collection session, the system was calibrated according to the manufacturer’s guidelines. Furthermore, to minimize potential integration-related drift, a reference resting posture was recorded at both the beginning and the end of each acquisition, allowing the system software to identify and compensate for any signal drift potentially occurring during the recording phase.

The 6MWT was performed along a 30 m-long, flat, straight indoor path marked by two rubber cones in a hard-surfaced hallway. After sensor setup and calibration of the motion-tracking system, participants began walking at a verbal start signal and continued back and forth along the path for six minutes, aiming to cover the greatest possible distance [28]. They were instructed to pivot briskly around the cones. An operator informed participants of the remaining time at one-minute intervals. At the end of the sixth minute, participants were asked to stop at their current location and remain in place.

Raw data were processed using dedicated software (MyoResearch v4.0, Noraxon Inc., Scottsdale, AZ, USA), and the variables of interest (i.e., heel-strike events and joint angle trajectories for hip and knee flexion–extension, and ankle dorsi-plantarflexion) were exported as ASCII files. These data served as input for a custom routine developed in the Matlab^®^ environment, which computed the PCI as well as inter- and intra-limb coordination parameters based on cyclogram geometric features, as described in detail below. Before calculating the parameters of interest, the same routine was used to remove the 180° turns occurring between consecutive 30-m steady-state walking segments. Specifically, turning segments were identified from the raw signal acquired by the inertial sensor positioned on the pelvis by analyzing the Euler yaw angle, which represents rotation around the vertical axis. The pelvis yaw signal was first converted into radians and phase-shifted to avoid discontinuities associated with angular wrapping, then converted back into degrees and smoothed using a Gaussian filter with a window length of 1500 samples. The absolute first derivative of the smoothed signal was subsequently calculated, and candidate turning events were identified as peaks separated by at least 2000 samples. Peaks were classified as turns when their amplitude exceeded an empirically defined threshold corresponding to one third of the difference between the maximum and mean values of the absolute derivative. Each detected turning event was then related to the heel-strike events to identify the gait cycles involved in the change of direction. The turning segment, together with the two gait cycles immediately preceding and following it, was excluded from the analysis to remove not only the actual rotation around the cone but also the acceleration, deceleration, and transitional gait patterns occurring before and after each turn, thereby ensuring that only steady-state straight-line walking data were retained for subsequent analyses. Finally, an average of 50 and 48 strides were retained for unaffected individuals and 47 and 46 strides for pwMS at minutes 1 and 6 of the test, respectively.

### Temporal measures of locomotor coordination: Phase coordination index

As previously described, the PCI is a metric that combines the accuracy and consistency of stepping phase generation relative to 180°, which represents ideal anti-phased left–right stepping [17]. After excluding turns, strides and steps were determined, and the leg with the longer average swing time was designated as the reference leg. For each stride of the reference leg, the relative timing of the contralateral heel-strike was used to estimate inter-limb phase. Accuracy was quantified as mean absolute difference between phase and 180°, normalized to 180° and expressed as a percentage, whereas consistency was defined as the coefficient of variation (CV) of phase values. These two components were normalized and summed to obtain the PCI. The interpretation of this parameter is straightforward, as lower values indicate better bilateral coordination (reference values for healthy individuals typically range between 2% and 4% [16, 29, 30]). This procedure was used to compute PCI on a minute-by-minute basis.

### Spatial measures of locomotor coordination: Cyclograms for inter-limb and inter-joint coordination analysis

**Fig. 1:**
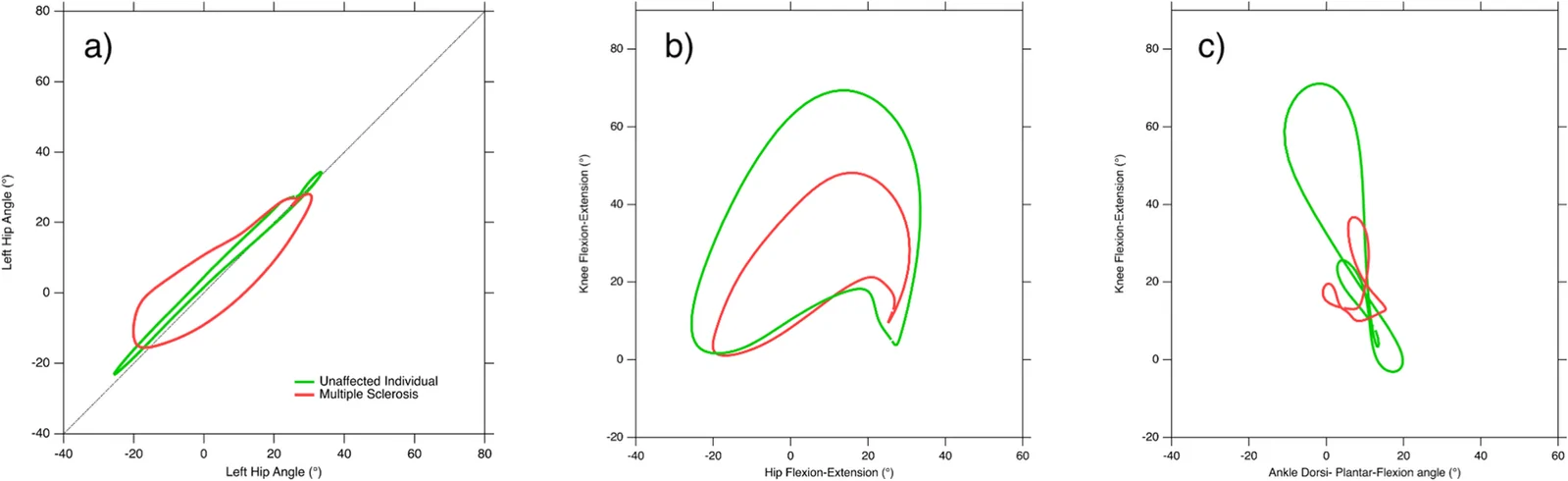
Examples of inter-limb and inter-joint cyclograms. From left to right: **a**) inter-limb cyclogram (hip joint), **b**) hip-knee inter-joint cyclogram, **c**) knee-ankle inter-joint cyclogram. The green curves refer to an unaffected individual, the red curves to an individual with MS

In order to quantify the inter-limb coordination, synchronized bilateral cyclograms were generated [31]. To this end, right and left limb angle values acquired during the gait cycle were used to build angle-angle diagrams for each joint of interest (i.e. hip, knee and ankle). Several geometrical features of cyclograms were then extracted as follows (see also Fig. 1 for a graphical explanation):

- Cyclogram area (degrees^2^) represents the area of the closed curve obtained from each angle-angle diagram [8]. Since a perfectly symmetrical gait is achieved when both left and right joints are positioned at the same angle for a certain time of the gait cycle (i.e. all the cyclogram points stand on a 45° line in the diagram and thus the area is null); the interpretation of this parameter is quite straightforward: that is, the smaller the area the more symmetrical the gait.
- Cyclogram orientation (degrees): this feature is identified by the absolute value of angle ϕ formed by the 45° line which corresponds to perfect interlimb symmetry and the orientation of the principal axis of inertia, which corresponds to the minimum moment of inertia of the cyclogram [31, 32]. Smaller values of this angle indicate higher interlimb symmetry.
- Trend Symmetry: this dimensionless parameter is calculated on the basis of eigenvector analysis on time-normalized right leg and left leg gait cycles ([33] for details of the mathematical procedure). Even in this case the interpretation of this parameter is quite simple; a 0 value indicates perfect symmetry, while asymmetry is more marked as the Trend Symmetry value increases.

Similarly, for the analysis of inter-joint coordination, the angle trends for hip, knee and ankle were processed with the same custom software to generate the hip-knee and knee-ankle diagrams for each limb and calculates the following parameters:

- Cyclogram area (degrees^2^): represents the area of the closed curve obtained by each angle-angle diagram [8]. Larger areas generally indicate a higher conjoint range of the angular movements experienced at the specified joints during a complete gait cycle [8, 32].
- Cyclogram perimeter (degrees): is the overall length of the closed trajectory. Although the perimeter generally increases with cyclogram area (especially in case of smooth diagrams), it is reported here primarily as an intermediate quantity used to compute the cyclogram dimensionless ratio (see below), a normalized descriptor of cyclogram shape.
- Cyclogram dimensionless ratio: is a parameter obtained by dividing the perimeter by the square root of the area and is intended to quantitatively represent the shape of the diagram. Generally speaking, cyclograms that appear more regular in shape (i.e., not particularly elongated towards a specific direction) are characterized by lower values of such ratio.

Examples of inter-limb and inter-joint cyclograms are shown in Fig. 1 for pwMS of different levels of disability and unaffected individuals.

For the inter-joint coordination analyses, the participant was considered the analytical unit. At each minute, one participant-level value was calculated for each inter-joint measure as the arithmetic mean of the corresponding values obtained from the right and left limbs. Bilateral averaging was used because limb side was not a prespecified factor and neither limb was designated a priori as the limb of interest. This procedure gave equal weight to both limbs and avoided treating correlated measurements from the same participant as statistically independent observations [33].

### Statistical analysis

To examine the effects of MS on gait coordination parameters, a two-way repeated-measures analysis of variance (RM-ANOVA) was performed with group (pwMS vs. unaffected individuals) as the between-subjects factor and time (minutes 1–6) as the within-subjects factor. The dependent variables were PCI as well as Inter-limb and Inter-joint parameters previously described. Effect sizes were quantified using partial eta‑squared (η²ₚ), calculated for each main effect and interaction as the proportion of variance explained relative to the effect plus its associated error variance. Significant main effects and interactions were further explored using Holm–Sidak corrected post-hoc tests. Because the six measurement points represented predefined repeated observations rather than a continuous time series, time was modelled as a within-subject categorical factor. In addition, polynomial contrast analyses were performed to investigate the presence of linear temporal trends where appropriate.

For the group of pwMS only, we also explored the existence of a relationship between a subset of inter-limb and inter-joint coordination parameters (i.e., PCI and cyclogram areas), the Multiple Sclerosis Walking Scale–12 score (MSWS-12, a patient-reported outcome measure specifically designed to assess the impact of MS on walking ability from the individual’s perspective [34, 35]) and the disability level (expressed through the EDSS score) using Pearson’s correlation coefficient *r*. in this case, since the MSWS-12 questionnaire was administered immediately before the 6MWT, for the coordination parameters we chose to employ the values calculated for the first minute only. Values of 0.1, 0.3, and 0.5 were assumed to be representative of small, moderate, and large correlations respectively according to guidelines [36]. All analyses were performed using the IBM SPSS Statistics v.20 software (IBM; Armonk; NY; USA).

## Results

All participants completed the 6MWT. On average, pwMS covered a significantly shorter distance compared with unaffected individuals (477.95 vs. 626.94 m, *p* < 0.001). The statistical analysis revealed a significant main effect of Group on gait speed [F(1,106) = 39.19, *p* < 0.001, η²ₚ = 0.27], indicating lower walking speed in pwMS compared with unaffected individuals throughout the 6MWT. A significant main effect of Minute was also observed [F(5,530) = 30.48, *p* < 0.001, η²ₚ = 0.22], indicating a progressive reduction in gait speed over the course of the test. In contrast, no significant Group × Minute interaction was detected [F(5,570) = 0.45, *p* = 0.814, η²ₚ = 0.004], suggesting that the temporal pattern of gait-speed changes was comparable between pwMS and unaffected individuals.

The values of the investigated coordination parameters for the first and last minute of the 6MWT are summarized in Table 2 (PCI and inter-limb cyclograms) and Table 3 (inter-joint cyclograms).

**Table 2:** Inter-limb coordination parameters in people with MS and unaffected individuals at the beginning and end of the 6MWT. Values are expressed as mean (SD)

		Multiple Sclerosis		Unaffected Individuals	
Parameter	Joint	Minute 1	Minute 6	Minute 1	Minute 6
Cyclogram area (degrees ^2^ )	**Hip**	208.95 (257.11) ^†^	231.32 (281.79) ^†^	109.73 (104.38)	143.71 (104.74)
Cyclogram orientation ϕ (degrees)		2.96 (3.19) ^†^	3.10 (3.10) ^†^	1.50 (1.20)	1.38 (1.09)
Trend Symmetry		2.79 (2.94) ^†^	2.90 (2.76) ^†^	1.53 (1.21)	1.38 (1.07)
Cyclogram area (degrees^2^)	**Knee**	350.23 (369.75) ^†^	381.36 (373.31) ^†^	238.02 (170.05)	248.49 (236.39)
Cyclogram orientation ϕ (degrees)		3.63 (4.39)	5.68 (15.27) ^†^	1.54 (1.09)	1.61 (1.16)
Trend Symmetry		3.48 (3.68)	5.87 (16.59) ^†^	1.56 (1.06)	1.60 (1.19)
Cyclogram area (degrees^2^)	**Ankle**	163.49 (165.86)	169.20 (168.08)	110.21 (86.06)	120.54 (95.64)
Cyclogram orientation ϕ (degrees)		4.88 (5.03) ^†^	4.46 (4.74)	2.93 (2.20)	3.06 (2.37)
Trend Symmetry		5.03 (5.80) ^†^	4.49 (5.02)	2.88 (2.01)	3.05 (2.18)
Phase Coordination Index (%)		8.24 (6.42) ^†^	9.22 (7.13) ^†^	3.55 (1.67)	4.68 (2.68)

The symbol ^†^ denotes a statistically significant difference with respect to unaffected individuals, within the same minute (*p* < 0.05)

**Table 3:** Inter-joint coordination parameters in people with MS and unaffected individuals at the beginning and end of the 6MWT. Values are expressed as mean (SD)

		Multiple Sclerosis		Unaffected Individuals		
Joint couple	Parameter	Minute 1	Minute 6	Minute 1		Minute 6
Hip-Knee	Cyclogram area (degrees^2^)	1669.19 (491.77)^†^	1669.12 (528.78)^†^	2171.56 (318.67)		2224.44 (337.20)*
	Cyclogram Perimeter (degrees)	181.73 (31.86)^†^	181.68 (33.35)^†^	211.99 (19.40)		212.16 (19.49)
	Dimensionless Ratio	4.52 (0.33)	4.54 (0.35)	4.56 (0.25)		4.51 (0.25)
Knee-Ankle	Cyclogram area (degrees ^2^)	452.01 (317.77)^†^	402.39 (266.93)* ^†^	791.64 (326.81)		693.96 (272.84)*
	Cyclogram Perimeter (degrees)	170.24 (28.36)^†^	169.90 (29.47)^†^	193.94 (14.43)		192.24 (14.58)
	Dimensionless Ratio	11.14 (7.00)	11.22 (5.18)	8.17 (5.18)		8.11 (2.85)

The symbol * denotes a statistically significant difference with respect to minute 1, within the same group (*p* < 0.001)

The symbol ^†^ denotes a statistically significant difference with respect to unaffected individuals, within the same minute (*p* < 0.001)

**Fig. 2:**
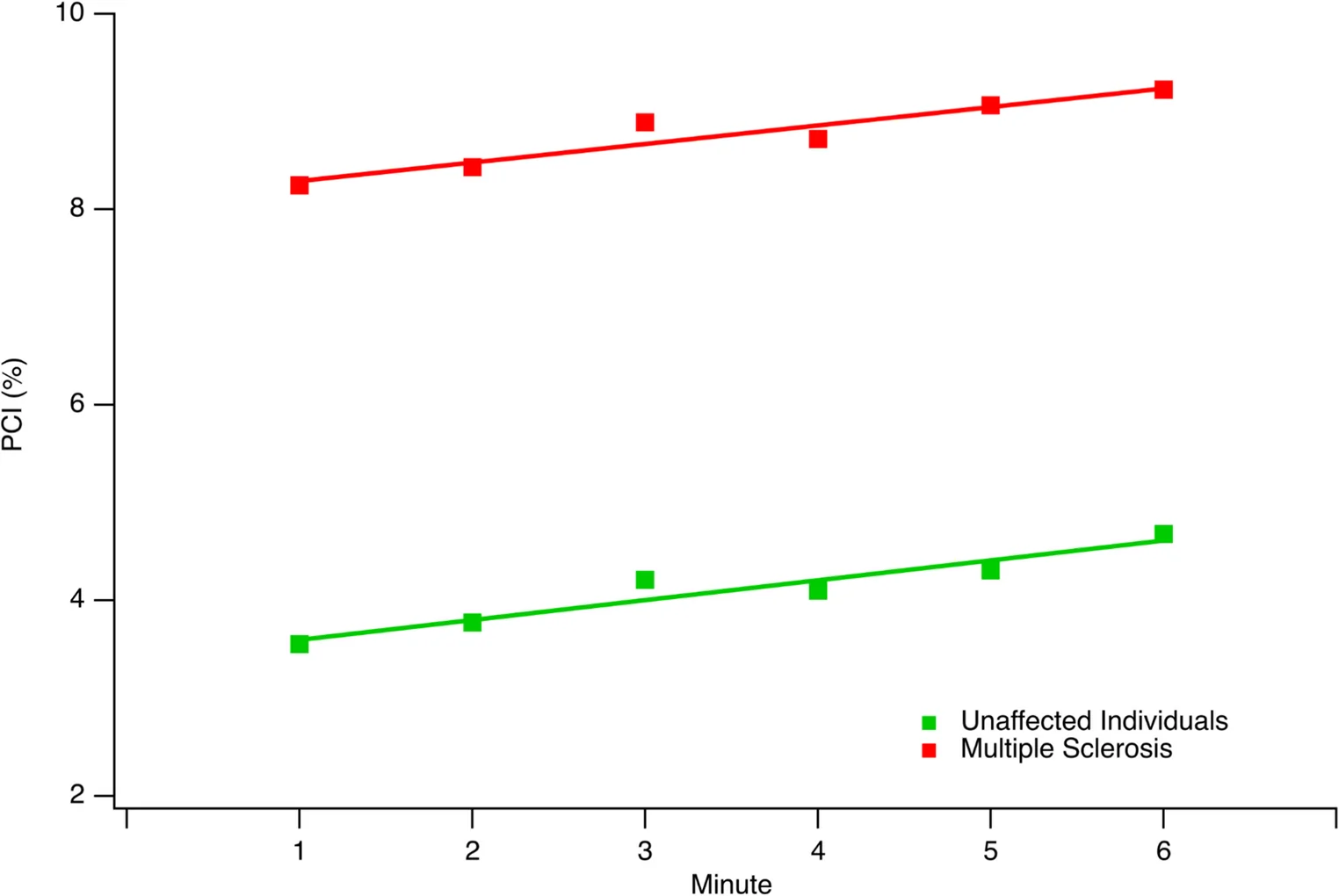
PCI values on a minute-by-minute basis for pwMS (red curve) and unaffected individuals (green curve) during the 6MWT. Lines represent fitted curves are included only as visual guides to illustrate the overall trend of the data and should not be interpreted as evidence of the strength or significance of the association

The statistical analysis revealed a significant main effect of Group on PCI [F(1,106) = 20.59, *p* < 0.001, η²ₚ = 0.16], indicating higher PCI values in the MS group compared with unaffected individuals. In contrast, no main effect of Minute [F(5,530) = 2.04, *p* = 0.071, η²ₚ = 0.02] or Group × Minute interaction [F(5,530) = 0.02, *p* = 1.000, η²ₚ < 0.001] was observed. However, an additional polynomial contrast analysis revealed a significant positive linear trend in PCI across the six minutes (*p* = 0.027), with no evidence of a different linear trend between groups. The corresponding minute-by-minute changes are shown in Fig. 2.

Regarding inter-limb coordination, a significant main effect of Group was observed at the hip joint for all considered parameters, including cyclogram area [F(1,106) = 5.41, *p* = 0.022, η²ₚ = 0.05], orientation [F(1,106) = 9.39, *p* = 0.003, η²ₚ = 0.08], and trend symmetry [F(1,106) = 8.34, *p* = 0.005, η²ₚ = 0.07]. The mean values of all three variables were higher in pwMS. No main effect of Minute or Group × Minute interaction was detected.

At the knee joint, a significant main effect of Group was found for cyclogram area [F(1,106) = 4.25, *p* = 0.042, η²ₚ = 0.09], orientation [F(1,106) = 10.47, *p* = 0.002, η²ₚ = 0.09], and trend symmetry [F(1,106) = 10.42, *p* = 0.002, η²ₚ = 0.09]. Again, no significant effect of Minute or Group × Minute interaction was observed.

Finally, at the ankle joint, a significant effect of Group was found for orientation [F(1,106) = 4.52, *p* = 0.036, η²ₚ = 0.04] and trend symmetry [F(1,106) = 4.20, *p* = 0.043, η²ₚ = 0.04], but not for cyclogram area [F(1,106) = 3.71, *p* = 0.057, η²ₚ = 0.03]. As in previous analyses, no main effect of Minute or Group × Minute interaction was detected.

**Fig. 3:**
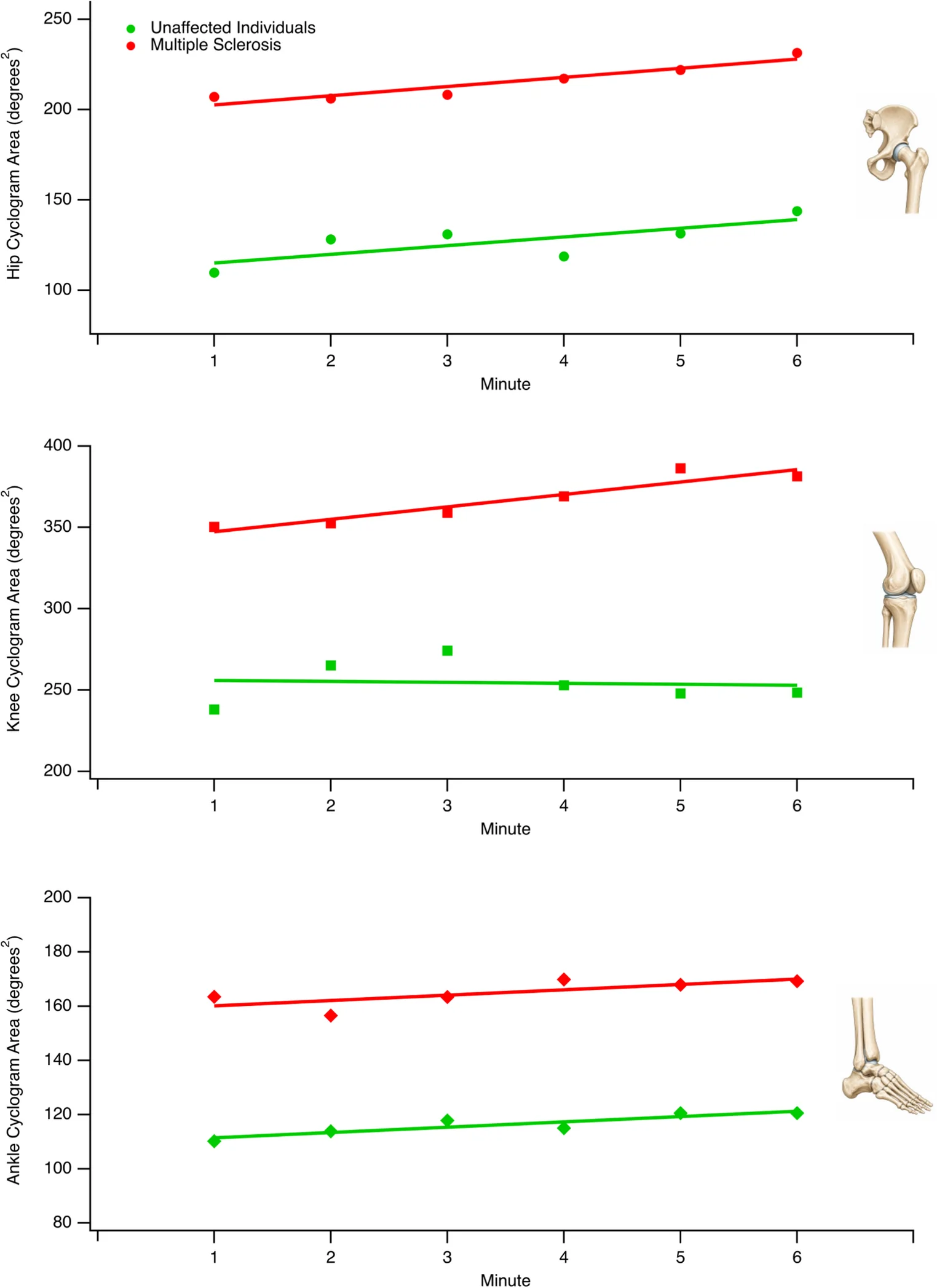
Values on a minute-by-minute basis of inter-limb cyclograms area for pwMS (red curve) and unaffected individuals (green curve) during the 6MWT. Top to bottom: hip, knee and ankle joints. Lines represent fitted curves included only as visual guides to illustrate the overall trend of the data and should not be interpreted as evidence of the strength or significance of the association

The average values of inter-limb cyclogram area during the 6MWT are illustrated in Fig. 3, from which it can be observed that similar patterns characterized both pwMS and unaffected individuals as regards hip and ankle joint. In contrast, at the knee joint, cyclogram area of pwMS remained relatively stable throughout the 6MWT.

As for inter-joint coordination, when considering the hip–knee joint couple, the statistical analysis revealed a significant main effect of Group on cyclogram area [F(1, 106) = 37.78, *p* < 0.001, η²ₚ = 0.26] and perimeter [F(1, 106) = 31.32, *p* < 0.001, η²ₚ = 0.23] but not of Minute. In this case, both parameters were significantly reduced in pwMS. Post hoc analysis further revealed that, only in unaffected individuals, the cyclogram area was significantly reduced at minute 6 compared with the first minute.

For the knee–ankle joint couple, significant main effects of Group [F(1, 106) = 31.99, *p* < 0.001, η²ₚ = 0.23] and Minute [F(5,530) = 23.83, *p* < 0.001, η²ₚ = 0.18] were detected for cyclogram area. The post hoc analysis showed that, in unaffected individuals, the cyclogram area was significantly reduced from minute 2 onward compared with the first minute, whereas in pwMS, this reduction occurred from minute 3 onward. Also, a main effect of Group only was observed for cyclogram perimeter [F(1, 106) = 24.36, *p* < 0.001, η²ₚ = 0.19], with values consistently higher in unaffected individuals at every minute. Lastly, regarding the dimensionless ratio parameter, the only statistically significant result involves the knee-ankle joint. Specifically, the analysis revealed a significant main effect of Group [F(1, 106) = 10.51, *p* = 0.0012, η²ₚ = 0.09], meaning that pwMS exhibit, for all minutes of the trial, a value higher with respect to unaffected individuals.

**Fig. 4:**
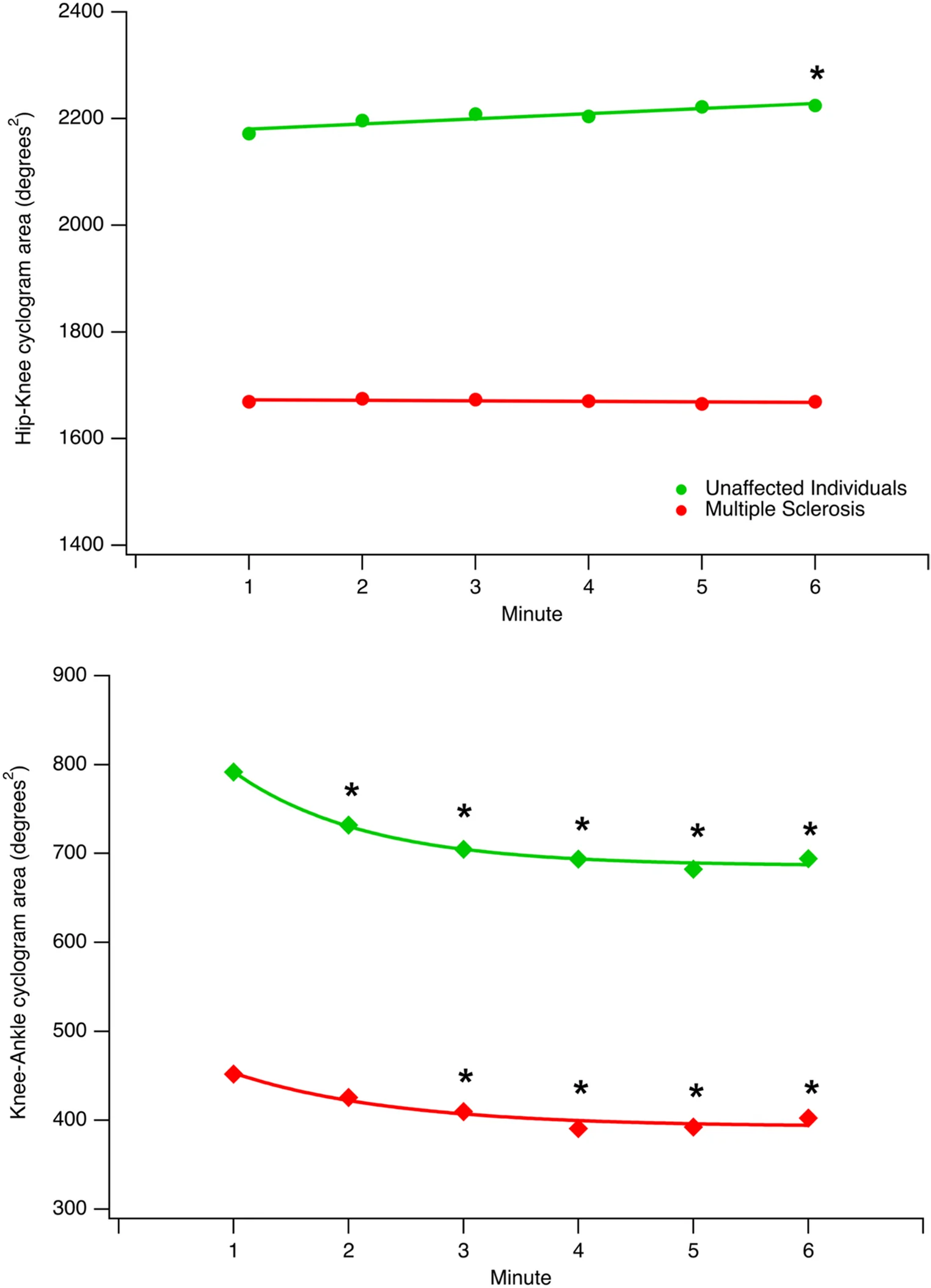
Values on a minute-by-minute basis of inter-joint cyclograms area for pwMS (red curve) and unaffected individuals (green curve) during the 6MWT. Top: hip-knee, bottom: knee-ankle joints. The symbol * denotes a statistically significant difference vs. minute 1. The fitted curves are included only as visual guides to illustrate the overall trend of the data and should not be interpreted as evidence of the strength or significance of the association

Figure 4 illustrates the changes in inter-joint cyclogram area for both joint couples during the test. While values for the hip–knee couple remained substantially stable throughout the trial, values decreased during the test for the knee–ankle couple, with slightly more pronounced changes in unaffected individuals, particularly during the first three minutes.

The results of the correlation analysis between inter-limb coordination parameters (PCI and cyclogram areas at hip, knee and ankle joints), inter-joint coordination parameters (hip-knee and knee-ankle cyclogram areas) and the MSWS-12 score are presented in Table 4. A moderate positive correlation was found between the PCI and the MSWS-12 score (*r* = 0.426, *p* < 0.001), while moderate-to-large negative correlations involved cyclograms area of both hip-knee and knee-ankle joint couples (*r* = −0.499 and *r* = −0.473 respectively, *p* < 0.001 in both cases).

**Table 4:** Pearson correlation coefficient between the gait coordination parameters (calculated at minute 1 of the 6MWT) and the perceived walking impairment (assessed through the MSWS-12 scale) in people with MS

		MSWS-12	EDSS
Inter-limb coordination	PCI	0.426**	0.509**
	Hip cyclogram area	0.137	0.111
	Knee cyclogram area	0.197	0.203
	Ankle cyclogram area	0.005	0.015
Inter-joint coordination	Hip-Knee cyclogram area	−0.499**	−0.526**
	Knee-Ankle cyclogram area	−0.473**	−0.481**

The symbol ** denotes statistical significance (*p* < 0.001)

## Discussion

The main aim of this study was to assess the presence and magnitude of changes in inter-limb and inter-joint coordination during a functional walking test (namely, the 6MWT) in pwMS by combining temporal and spatial locomotor coordination parameters derived from discrete spatiotemporal measures (PCI) and waveform-based methods (cyclograms). This information is crucial for the analysis of motor dysfunctions associated with MS, as poor coordination directly affects symmetry, which has been recognized as a key domain influencing gait quality and efficiency [6], alongside pace, rhythm, variability, and complexity. Coordination deficits also disrupt inter-joint coupling, producing less smooth and less efficient movement patterns and increasing the energetic cost of walking [37]. In addition, poor coordination can compromise dynamic stability and balance, increasing the risk of falls [38], and may promote the use of compensatory strategies that further alter normal gait mechanics.

First, consistent with previous studies, our results confirm that both inter-limb and inter-joint coordination are deteriorated in pwMS when compared to unaffected individuals, as observable from the significant inter-group differences detected in most investigated parameters since the early stages of the 6MWT. As regards the inter-limb coordination, it is noticeable that values of PCI and cyclogram parameters calculated during the first minute of test are in good agreement, even from a quantitative point of view, with those reported in previous studies on pwMS (for PCI see [15–17], for cyclograms see [22]) despite some heterogeneity in terms of clinical features of the participants, equipment and test conditions, thus demonstrating the robustness of both approaches. Similar considerations involve the inter-joint coordination, particularly as regards the cyclograms area and perimeter, which are systematically reduced in pwMS since minute 1, thus indicating a reduced dynamic range of motion and less ability to modulate joint interactions across the different phases of the gait cycle [11].

Our initial hypotheses regarding the detrimental effects of prolonged walking on coordination parameters were only partly confirmed. In fact, although a deterioration of temporal and spatial inter-limb and inter-joint coordination parameters over the course of the 6MWT was observed, only a few parameters showed statistically significant changes by the end of the trial. More importantly, despite substantial differences in absolute values, that is with pwMS consistently exhibiting poorer PCI and cyclogram features, the variations occurring during the 6MWT were either similar across groups or, in some cases, even more pronounced in unaffected individuals.

Focusing on inter-limb coordination, our data indicate that the PCI value at the end of the 6MWT is 53% (unaffected individuals) and 32% (pwMS) higher than that calculated during the first minute of the test. In this regard, it is worth noting that although similar experiments have previously been conducted on pwMS [17, 20], the magnitude of change on a minute-by-minute basis was not reported. Moreover, no control group was included, making direct comparison with our findings difficult. Nevertheless, our results reasonably support two facts: (1) pwMS exhibit higher baseline PCI values, with the magnitude partly dependent on disability level (as also confirmed by our correlation analysis), and (2) a general linear increase in PCI during the 6MWT is to be expected, as demonstrated by the polynomial contrast analysis which demonstrated a significant positive linear trend across the six minutes of the test, thus indicating that PCI increased progressively over time despite the absence of a significant overall time effect.

When inter-limb coordination is analyzed from a spatial perspective, a group effect remains evident at most time points, particularly at the hip and knee joints; however, no significant time effect was detected. Across the six predefined time points, similar patterns were observed in both groups for the investigated parameters at the hip and ankle joints, although the magnitude of the changes differed. For example, the cyclogram area at both joints increased by approximately 30% from minute 1 to minute 6 in unaffected individuals, compared with an increase of approximately 10% in pwMS. In contrast, at the knee level, values observed in the pwMS group seem to remain practically unaltered throughout all the test. Taken together, these findings suggest that, in pwMS, the 6MWT induces alterations in inter-limb coordination that are similar to, if not smaller than, those observed in unaffected individuals. These results likely reflect a combination of baseline impairment and limited adaptive capacity in pwMS. The former are likely to be due to a combination of the following factors: reduced efficiency in the neural communication pathways between the two cerebral hemispheres, particularly as regards the fiber bundle connecting the primary motor cortices [16], microstructural damage of the spinal cord [39] and altered integrity of the long ascending and descending myelinated fiber tracts of cortical, cerebellar and brainstem systems [40]. Moreover, during the prolonged walking test, additional changes are likely to occur due to the onset of fatigue, which is known to degrade motor control and temporal coordination [20].

However, the smaller relative increase observed in pwMS compared with unaffected individuals suggests the presence of a ceiling effect. This phenomenon is unlikely to be explained by a single mechanism. On the one hand, people with MS walked consistently at a lower speed throughout the 6MWT, and gait speed is known to influence both temporal and spatial coordination. Walking at a slower speed may reduce the range of available coordination strategies and partly limit the magnitude of further detectable changes. On the other hand, the marked baseline impairment in coordination observed in pwMS strongly suggests that disease-related neuromotor deficits also play a major role. Altered interhemispheric communication, spinal cord damage, and impaired integrity of corticospinal and cerebellar pathways are all likely to constrain the ability to further adapt locomotor coordination during prolonged walking. Therefore, the apparent ceiling effect probably reflects the combined influence of reduced walking speed and impaired neuromotor adaptability rather than either factor alone.

Finally, the distinct behavior observed at the knee joint, where only unaffected individuals show significant worsening of coordination parameters, may be explained by joint-specific control mechanisms. The knee plays a critical role during the stance phase in load acceptance and body weight support, as well as in ensuring adequate foot clearance during the swing phase. Thus, we can hypothesize that while in unaffected individuals this joint may be more sensitive to fatigue-induced changes in neuromuscular control, the presence of compensatory strategies typical of MS (e.g., increased reliance on proximal joints or muscular co-contraction to enhance stability [41]) may limit observable changes, effectively masking further deterioration in coordination.

As regards inter-joint coordination, here investigated only in terms of spatial measures (i.e., cyclograms), the most relevant alterations observed during the 6MWT involve the knee–ankle joint couple. Interestingly, the magnitude of reduction of knee–ankle cyclogram area across the whole test was similar in unaffected individuals and pwMS, despite a more marked change between minutes 1 and 2 in the former. This finding suggests an overall comparable impact of fatigue on distal coordination. Generally speaking, this result is not surprising, considering the pivotal role played by the ankle joint in propulsion and foot clearance. Indeed, it is reasonable to hypothesize that fatigue-related impairments at this joint, manifesting as reduced plantarflexion at push-off and altered muscle activation patterns (indicative of diminished power generation and changes in neuromuscular timing [42]) lead to a reduced excursion and a tighter coupling with the knee, thereby decreasing the cyclogram area.

However, the mechanisms underlying this behavior likely differ between groups. In unaffected individuals, the observed changes may reflect a fatigue-induced reorganization of motor control, characterized by a shift toward more constrained and stability-oriented coordination patterns. In contrast, in pwMS, distal coordination is already severely altered at baseline due to disease-related impairments (as previously reported [11]). Consequently, fatigue induces comparatively smaller additional adaptations, similarly to what has been described for inter-limb coordination. From this perspective, the comparable magnitude of change observed between groups may indicate a convergence toward a shared, fatigue-driven motor strategy that prioritizes stability over flexibility at the distal joints, rather than reflecting similar underlying neuromuscular mechanisms.

Some limitations of this study should be acknowledged. First, due to the relatively small size of the pwMS group, we were unable to perform appropriate stratified analyses to clarify the role of important factors that may influence gait kinematics and coordination, such as disability level, sex, and fatigability. Second, gait speed was not experimentally controlled and differed both between groups and over time; therefore, given the potentially heterogeneous pacing strategies adopted across groups and among participants within each group, our analysis cannot disentangle changes in gait coordination occurring over the course of the test from those associated with concurrent changes in gait speed. In this regard it should be noted that increasing gait speed can paradoxically improve inter-limb coordination, potentially through the recruitment of optimized subcortical motor circuits [18]. Therefore, a more refined analysis incorporating the speed profiles of pwMS could facilitate the identification and clustering of individuals who adopt different strategies during the 6MWT, particularly those who increase their speed over the course of the test and, consequently, may experience a reduction in PCI. Third, although gait analysis was restricted to straight walking segments, the repeated turning required by the 6MWT may have contributed to overall fatigue and sensorimotor load [43]. Such effects, which were not directly quantified, could differentially influence coordination strategies in pwMS and unaffected individuals. A further limitation concerns the modelling of the temporal dimension, as time was treated as a categorical within-subject factor rather than as a fully sequential variable. Although participant-specific nonlinear regression could provide a more detailed characterization of individual temporal trajectories, it was not adopted because of its greater analytical complexity and the resulting reduction in the clarity and interpretability of the findings.

## Conclusion

This study provides a comprehensive characterization of gait coordination during the 6MWT in pwMS by jointly examining inter‑limb and inter‑joint coordination through the integration of temporal and spatial measures. By combining a discrete timing‑based index (PCI) with waveform‑based metrics derived from cyclograms, it was possible to capture complementary aspects of locomotor coordination that are not accessible when using a single methodological approach. This multimodal framework represents a key novelty of the present work and offers a deeper insight into the complexity of gait control alterations associated with MS under prolonged walking conditions.

Our findings confirm that both temporal and spatial coordination are substantially compromised in pwMS compared with unaffected individuals from the earliest stages of the 6MWT, highlighting coordination as a core domain of gait dysfunction in this population. However, despite clear baseline impairments, prolonged walking induced only limited additional deterioration in coordination in pwMS, whereas unaffected individuals exhibited more pronounced fatigue‑related changes. This pattern supports the hypothesis of a ceiling effect in MS, where pre‑existing neuromotor deficits constrain the capacity for further adaptive or maladaptive reorganization during sustained walking.

From a clinical and rehabilitative perspective, these results have several relevant implications. First, the observed dissociation between baseline coordination deficits and fatigue‑related changes suggests that rehabilitation strategies should not only aim to improve endurance, but also explicitly target coordination quality and adaptability. Second, the sensitivity of cyclogram‑based measures to inter‑joint coupling, particularly at the knee–ankle level, indicates that distal coordination may represent a promising target for interventions aimed at enhancing propulsion efficiency and stability during prolonged walking. Finally, the complementary information provided by temporal and spatial coordination metrics may help clinicians tailor rehabilitation programs more precisely, monitor responses to therapy, and identify individuals who rely on compensatory strategies rather than adaptive motor control.

In summary, the present study highlights the value of integrating temporal and spatial coordination measures to obtain a more complete and clinically meaningful assessment of gait dysfunction in multiple sclerosis. Such an approach may support the development and evaluation of rehabilitation interventions specifically designed to improve coordination, ultimately contributing to better walking performance, reduced fatigability, and enhanced mobility in daily life for people with MS.

## Data Availability

The datasets generated during the current study are available from the corresponding author upon reasonable request.
